# Ultraslim catheter-type peroral cholangioscope-assisted endoscopic transpapillary gallbladder drainage for acute cholecystitis

**DOI:** 10.1055/a-2462-2162

**Published:** 2024-12-12

**Authors:** Noriyuki Hirakawa, Atsushi Sofuni, Takayoshi Tsuchiya, Ryosuke Tonozuka, Shuntaro Mukai, Takao Itoi

**Affiliations:** 1 13112 Department of Gastroenterology and Hepatology, Tokyo Medical University, Tokyo, Japan

Endoscopic transpapillary gallbladder drainage (ETGBD) is clinically effective for acute cholecystitis but is technically challenging 1 2 . In addition, the shape and bifurcation of the cystic duct represent a technical limitation and difficulty of this method 2 3 4 . Recently, an ultraslim catheter-type peroral cholangioscope (UC-POCS, DRES Slim Scope; Japan Lifeline Co., Ltd, Tokyo, Japan) has been developed and used clinically 5 . The UC-POCS consists of a catheter-type sheath (outer diameter 2.3 mm) with two independent lumens and a high-resolution digital camera. The device can be used as a catheter for common bile duct (CBD) cannulation ( Fig. 1 ). Herein, we report a case in which UC-POCS-assisted ETGBD was performed for acute cholecystitis.

**Fig. 1:**
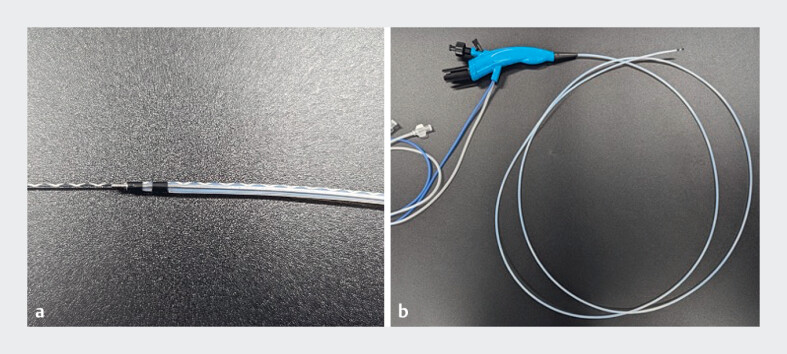
The ultraslim catheter-type peroral cholangioscope device consists of: **a** a catheter-type sheath (outer diameter 2.3 mm); **b** two independent lumens and a high-resolution digital camera.

A 61-year-old woman with type 2 diabetes mellitus visited our clinic with chills and right upper quadrant abdominal pain. Abdominal computed tomography showed a prominent enlarged gallbladder with edematous wall thickening and she was diagnosed as having moderate acute cholecystitis ( Fig. 2 ). Urgent cholecystectomy was indicated but had to be abandoned due to concurrent diabetic ketosis. We therefore attempted ETGBD.

**Fig. 2:**
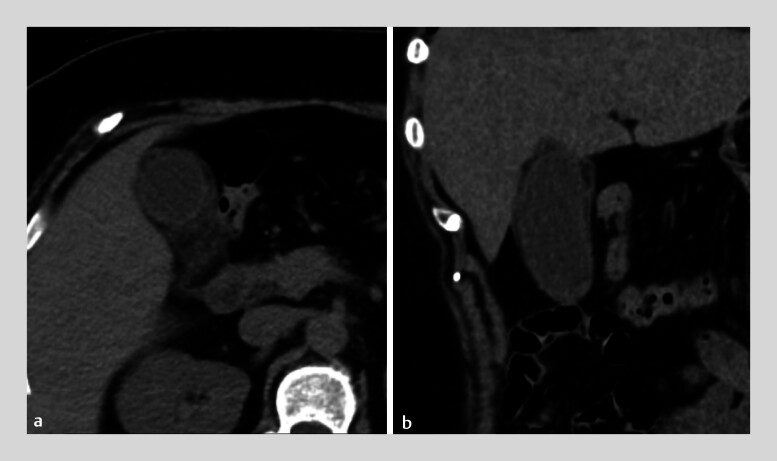
Abdominal computed tomography showing a prominent enlarged gallbladder with edematous wall thickening.

First, the CBD was successfully cannulated without any difficulty using the novel catheter, but the cystic duct could not be identified on subsequent cholangiography ( Video 1 ). Therefore, the POCS mounted in the catheter was used to look inside the CBD and could identify the bifurcation of the cystic duct. Additional cholangiography showed an impacted stone at this location. The guidewire was manipulated under fluoroscopy and was successfully inserted into the gallbladder. Following the removal of the UC-POCS, ETGBD with a 5-Fr naso-drainage tube was successfully achieved with no adverse events.

Ultraslim catheter-type peroral cholangioscope-assisted endoscopic transpapillary gallbladder drainage for acute cholecystitis.Video 1

Endoscopy_UCTN_Code_TTT_1AR_2AB

## Notes

Endoscopy E-Videos https://eref.thieme.de/e-videos
*E-Videos* is an open access online section of the journal *Endoscopy* , reporting on interesting cases and new techniques in gastroenterological endoscopy. All papers include a high-quality video and are published with a Creative Commons CC-BY license. Endoscopy E-Videos qualify for HINARI discounts and waivers and eligibility is automatically checked during the submission process. We grant 100% waivers to articles whose corresponding authors are based in Group A countries and 50% waivers to those who are based in Group B countries as classified by Research4Life (see: https://www.research4life.org/access/eligibility/ ). This section has its own submission website at https://mc.manuscriptcentral.com/e-videos .
